# Genotyping and detection of common avian and human origin-influenza viruses using a portable chemiluminescence imaging microarray

**DOI:** 10.1186/s40064-016-3482-9

**Published:** 2016-10-25

**Authors:** Yingjie Zhang, Qiqi Liu, Dou Wang, Suhong Chen, Xiaobo Wang, Shengqi Wang

**Affiliations:** 1 Department of Pharmacy, 210th Hospital of the Chinese People’s Liberation Army, Dalian, 116021 People’s Republic of China; 2 Postdoctoral Research Workstation, 210th Hospital of the Chinese People’s Liberation Army, Dalian, 116015 People’s Republic of China; 3 Key Laboratory of New Molecular Diagnosis Technologies for Infectious Diseases, Institute of Radiation Medicine, Academy of Military Medical Sciences, Beijing, 100850 People’s Republic of China; 4 Key Laboratory of New Molecular Diagnosis Technologies for Infectious Diseases of Beijing, Beijing, 100850 People’s Republic of China

**Keywords:** Avian-origin influenza viruses, Human-origin Influenza viruses, Chemiluminescence, Microarray

## Abstract

**Background:**

Influenza viruses are divided into three types, A, B, and C. Human influenza A and B viruses can cause seasonal epidemics, but influenza C causes only a mild respiratory illness. Influenza A virus can infect various host species. In 2013, human-infectious avian influenza A (H7N9) was first reported in China. By the second week of 2014, there were 210 laboratory-confirmed human cases in the country, and the mortality rate eventually reached 22 %. Rapid and accurate diagnosis of influenza viruses is important for clinical management and epidemiology.

**Methods:**

In this assay, a cost-effective chemiluminescence (CL) detection oligonucleotide microarray was developed to genotype and detect avian influenza A (H7N9), avian influenza A (H5N1), 2009 influenza A (H1N1), seasonal influenza A (H1N1), and seasonal influenza A (H3N2). Influenza A viruses and influenza B viruses were also generally detected using this microarray.

**Results:**

The results of detection of 40 cultivated influenza virus strains showed that the microarray was able to distinguish the subtypes of these influenza viruses very well. The microarray possessed similar or 10 fold higher limit of detection than the real-time RT-PCR method. Sixty-six clinical swab samples were detected using this microarray and verified with real time RT-PCR to evaluate the efficiency of this microarray for clinical testing.

**Conclusions:**

A reliable CL detection oligonucleotide microarray had been developed to genotype and detected these influenza viruses.

## Background

Influenza viruses are divided into three types, A, B, and C (WHO 1980). Human influenza A and B viruses always cause seasonal epidemics, but influenza C causes only a mild respiratory illness. Influenza A viruses are further divided into various subtypes based on hemagglutinin (HA) and neuraminidase (NA). Currently, seventeen serotypes of hemagglutinin (H1–H17) and ten serotypes of neuraminidase (N1–N10) of influenza A virus have been identified in mammalian and avian species (Fouchier et al. 2005; Zhu et al. 2013). Influenza B viruses are broken down into B/Yamagata and B/Victoria lineages (Zhang et al. 2012). They are not divided into subtypes.

Influenza A virus can infect various host species. The greatest diversity of subtypes is found in wildfowl. The ease with which influenza A can recombine causes viral epidemics to spread across different species (dos Reis et al. 2011). There have been several influenza A pandemics during the past 100 years: influenza A (H1N1) caused Spanish flu in 1918, causing 50 million deaths (Taubenberger and Morens 2006). Another influenza A (H2N2), caused Asian flu in 1957, with one million deaths (Xu et al. 2010). Influenza A (H3N2) caused Hong Kong flu in 1968, with another one million deaths (Viboud et al. 2005). A novel influenza A first noted in 2009, H1N1 caused the swine flu in 2009, with 0.28 million deaths (Dawood et al. 2012). There have also been small-scale epidemics, such as the avian influenza virus A (H5N1) outbreak in Hong Kong in 1997, in which 18 individuals were infected and 6 died (WHO 2004). In addition to the avian influenza virus A H5N1, highly pathogenic avian influenza virus A H5N2 (Yang et al. 2015; Zhan et al. 2012), avian influenza virus A H5N5 (Gu et al. 2011), H5N6 (Li et al. 2016; Qi et al. 2014), H5N8 (Wang et al. 2016; Zhao et al. 2013), and H5N9 (Yu et al. 2015) have also been isolated or confirmed from avian, mice, dog, or humans in China since 2000. It is noteworthy that the first human avian influenza A H5N6 infections were reported in Sichuan province (Nations and F.a.A.O.o.t.U. 2014) and caused 14 laboratory-confirmed human cases (including six deaths) by 9 May 2016 (Organization 2016). In 2013, human-infectious avian influenza A (H7N9) was reported in China (Gao et al. 2013b). By the second week of March 2016, there had been 752 laboratory-confirmed human cases in China, including at least 295 deaths (WHO 2016a, b). The clinical manifestations, severity, and mortality rates of human-infectious avian influenza A (H7N9) were similar to those of avian influenza A (H5N1) and 2009 influenza A (H1N1) but quite different from seasonal influenza (Gao et al. 2013a). Many studies have shown that it is more effective to reduce the severity of the infection when administration of NA inhibitor within 48 h of the onset of influenza symptoms than after 48 h (Hayden et al. 1997; McGeer et al. 2007; Monto et al. 1999). For these reasons, rapid and accurate diagnosis influenza viruses are important to clinical management and epidemiological.

Some molecular diagnostic technologies have been used to diagnose and subtype influenza viruses: antigens and antibody detection (Duman et al. 2013; He et al. 2013), real-time PCR (Choi et al. 2013; Dawood et al. 2009; Gao et al. 2013a; Hackett et al. 2014; Poon et al. 2009; Templeton et al. 2004), sequencing (Deng et al. 2011; Ghedin et al. 2011; Rutvisuttinunt et al. 2013), and microarray (Gall et al. 2009; Han et al. 2008; Heil et al. 2010; Ryabinin et al. 2011). In the assay described here, a chemiluminescence (CL) detection oligonucleotide microarray was developed and used to detect avian influenza A (H7N9), avian influenza A (H5N1), 2009 influenza A (H1N1), seasonal influenza A (H1N1), and seasonal influenza A (H3N2) by genotyping. The microarray also detected influenza A and influenza B viruses in all cases. The efficiency of this CL detection strategy was investigated in actual samples and the results were compared to those of real-time PCR methods.

## Methods

### Specimen collection and processing

The six clinically isolated strains of avian influenza A (H7N9) were obtained from the Centers for Disease Control (CDC) of Zhejiang Province. The other cultivated influenza virus strains were obtained from the National Institutes for Food and Drug Control. Clinical throat swab samples were collected from patients suspected of having infected influenza in the 307th Hospital of the Chinese People’s Liberation Army for whom it was not possible to determine whether they had taken medication. Non-influenza respiratory viruses were identified and obtained from the National Institute for Viral Disease Control and Prevention and the 302nd Hospital of the Chinese People’s Liberation Army. Lysate had already been added to all these viral samples before they were acquired for the present study. Total RNAs were extracted using the TIANamp Virus RNA Kit as described in the manufacturer’s protocol (TIANGEN Biotech Beijing Co., Ltd.) and stored at −70 °C until use.

### Primer and probe design

HA and NA gene FASTA sequences of avian influenza H7N9 virus, NA gene FASTA sequences of avian influenza H5N1 virus, 2009 influenza A (H1N1), seasonal influenza A (H1N1), influenza A (H3N2), matrix protein (M) gene of influenza A virus, and nonstructural protein (NS) gene of influenza B virus were downloaded from NCBI’s influenza nucleotide database. The sequences were then aligned using AlignX (a component of the Vector NTI Advance 10.3.0) to compare homology. Genotyping primers for these subtypes of influenza A were designed using Primer Premier 5 (PREMIER Biosoft International, USA) and they served as the consensus sequences of the HA and NA genes. Universal primers for influenza A virus and influenza B virus were designed and served as the consensus sequences of the relatively well-conserved M and NS genes. Microarray probes ranging from 20 to 40 nt were designed to detect influenza A virus generally, influenza B virus generally, and these subtypes of influenza A virus. Internal standard primers and probes based on the sequences of *Homo sapiens* ribonuclease P were designed to monitor specimen extraction, RT-PCR amplification, and microarray hybridization. This marker has been described as a reliable internal positive control marker in several publications (Dare et al. 2016; Fan et al. 2014). All the primers and probes were confirmed using BLAST program of NCBI and then synthesized using Sangon Biotech Co., Ltd. (Shanghai). The primers and probes are shown in Tables 1 and 2.

**Table 1:** Primer sequences for microarray

Primer	Sequence (5′–3′)	Target	Gene	Location	Reference^a^
NA-1Sa	GTTCTATGCTCTCAGCCAAGG	H7N9	NA	378–398	CY146910
NA9r-B-4	GCATTGTTGTTTGGTCCTGATATAC	H7N9	NA	569–593	CY146910
H7F1	AGACCTCGGTCAATGYGG	H7N9	HA	201–218	CY146908
H7R2-B	TTCACGAATTTCCCAGGATAACA	H7N9	HA	313–335	CY146908
NF11	CAAGAGTCTGAATGTGCATG	H5N1	NA	699–718	AM183680
NR22B	GGATCCCAAATCATTTCAAA	H5N1	NA	1134–1153	AM183680
F7	GTGTGTGCAGGGATAACTGG	PH1N1	NA	889–908	CY081570
R4aB	ATTAGGGCGTGGATTGTCTC	PH1N1	NA	988–1007	CY081570
NF12	CAAGAGTCTGAATGTGTCTG	H1N1	NA	699–718	AJ006954
NR21B	GGATCCCAAATCATCTCAAA	H1N1	NA	1131–1150	AJ006954
NF5	CTGACCAACACCACCATA	H3N2	NA	221–238	CY091836
NR5B	CATCAATAGGGTCCGATA	H3N2	NA	482–499	CY091836
MF2	GGCCCCCTCAAAGCCGAGAT	Influenza A	M	77–96	HQ664927
MR2B	CAAAGCGTCTACGCTGCAGT	Influenza A	M	244–263	HQ664927
FBF1	ATGGCCATCGGATCCTCAACTCACTC	Influenza B	NS	737–762	CY099917
FBR1B	TCATGTCAGCTATTATGGAGCTGTT	Influenza B	NS	956–980	CY099917
RP-F4	TGGGATCATGTTAAGTAGAAGTAGC	Human	RNase P	1821–1854	NM_006413
RP-R4B	CTCCATTGTTTTAGAGCCCTTAC	Human	RNase P	1882–1904	NM_006413
RP-F1	TGCGGGTTGGAGAAAATACA	Human	RNase P	1964–1983	NM_006413
RP-R1B	GGAGGCTGAGGCAGGAGAAT	Human	RNase P	2086–2105	NM_006413
RP-F	AGATTTGGACCTGCGAGCG	Human	RNase P	50–68	NM_006413
RP-RB	GAGCGGCTGTCTCCACAAGT	Human	RNase P	95–114	NM_006413

^a^The numbers are NCBI accession codes

**Table 2:** Probe sequences for microarray

Probe	Sequence (5′–3′)^b^	Target	Gene	Location	Reference^a^
H7-a	ACTGGACCACCCCAATGTGACCAATTCCTAGAATTT	H7N9	HA	235–270	CY146908
H7-b	ATAGGACCTCCCCAATGCGATCAATTTCTGGAGTTT	H7N9	HA	235–270	CY146908
N9	GGAAACACTCAAACGGAACAATACACGATAGGTCCCAGTA	H7N9	NA	413–452	CY146910
PH1N1	GGCTCGAATCGACCGTGGGT	PH1N1	NA	912–931	CY081570
H1N1	CAATCCAGTGACTGTTGATGGAGCAA	H1N1	NA	1025–1050	AJ006954
H3N2	TAACATTACAGGATTTGCACCTTTTTC	H3N2	NA	295–321	CY091836
H5N1-a	CAAATAGGCCATGGGTATCTTTCAATC	H5N1	NA	856–882	AM183680
H5N1-b	GTGTCCCCTAACGGGGCATATGGGG	H5N1	NA	972–996	AM183680
A	CTCATGGAATGGCTAAAGACAAGACCAA	Influenza A	M	149–176	HQ664927
B	AATGAAGGACATTCAAAGCCAATTCGAGCAGCTGAAACTGCG	Influenza B	NS	775–816	CY099917
RP-P	TTCTGACCTGAAGGCTCTGCGCGCG	Human	RNase P	71–95	NM_006413
RP-P1	CATGAACCCAGGAGGCGGAGCTTGC	Human	RNase P	1995–2019	NM_006413
PR-P4	ACATGCATTTATGCAATATTAATGT	Human	RNase P	1859–1883	NM_006413
(T)_20_	TTTTTTTTTTTTTTTTTTTT	Quality control^c^			

^a^The numbers are NCBI accession codes

^b^A repeat sequence of (T)_12_ with an amino-labeled 3′-end was connected to the 3′-end of all the probes

^c^A repeat sequence of (T)_20_ with an amino-labeled 3′-end and biotin-labeled 5′-end was used for quality control during microarray

### Microarray preparation

A repeat sequence of (T)_12_ with an amino-labeled 3′-end was connected to the 3′-end of all the probes so that it could be fixed to the aldehyde-chip surface.

**Fig. 1:**
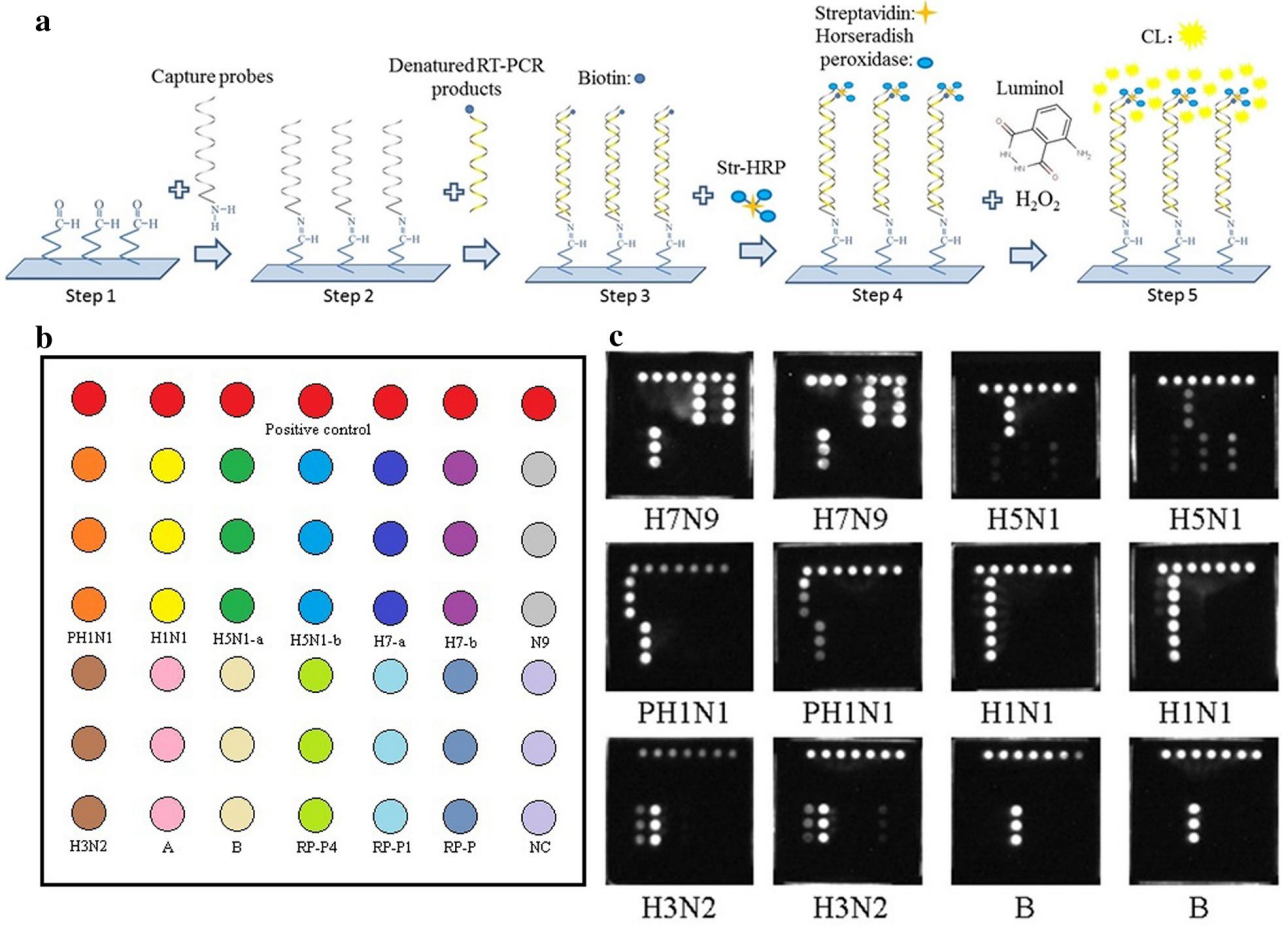
Microarray layout and CL detection results of parts of cultivated influenza viruses. **a** Working principle of this CL imaging DNA hybridization method. Steps 1–2 showed that capture probes were fixed to the aldehyde-chip surface. Step 3 showed that the denatured RT-PCR products were hybridized on the capture-chip. Steps 4–5 showed the CL detection principle. Biotin was incorporated into reverse strand on the RT-PCR amplification. Then, HRP modified streptavidin was bound and CL signal was generated by catalysed substrates. **b** Microarray layout. Capture probes were spotted in triplicate in col. The sequences of (T)_20_ were repeated seven times for quality control. **c** CL detection results of parts of cultivated influenza viruses. These cultivated influenza virus strains of influenza virus were derived from the National Institutes for Food and Drug Control and CDC of Zhejiang Province. The results showed that the microarray was able to distinguish the subtypes of avian influenza A (H7N9), avian influenza A (H5N1), 2009 influenza A (H1N1), seasonal influenza A (H1N1), influenza A (H3N2), and influenza B virus very well

A repeat sequence of (T)_20_ with a biotin-labeled 5′-end and an amino-labeled 3′-end served as a quality control probe. The probes were used at 50 μM final concentration. They were spotted and repeated three times in the vertical direction on the surface of the aldehyde-chip using uniform proportional printing buffer as described in previous studies (Zhang et al. 2013). The quality control probes were spotted and repeated seven times in the horizontal direction (Fig. 1b).

### RT-PCR amplification

The primers were used in three different RT-PCR systems. Each RT-PCR was performed in a 25 μl reaction volume containing 12.5 μl of 2× One Step Buffer, 1.0 μl of PrimeScript One-step Enzyme Mix (DRR055A, Takara Biotechnology (Dalian) Co., Ltd.), 5 μl of total RNA template, and specific primer mix. RT-PCR system A amplified novel avian influenza A (H7N9) and avian influenza A (H5N1). It contained primer H7F1 0.16 μM, H7R2-B 0.8 μM, NA-1Sa 0.12 μM, NA9r-B-4 0.6 μM, NF11 0.1 μM, NR22B 0.5 μM, RP-F4 0.08 μM, and RP-R4 0.4 μM. RT-PCR system B amplified 2009 influenza A (H1N1), seasonal influenza A (H1N1), and influenza A (H3N2). It contained primer F7 0.1 μM, R4aB 0.5 μM, NF5 0.1 μM, NR5B 0.5 μM, NF12 0.1 μM, NR21B 0.5 μM, RP-F1 0.1 μM, and RP-R1B 0.5 μM. RT-PCR system C amplified influenza A and B viruses. It contained primer MF2 0.1 μM, MR2B 0.5 μM, FBF1 0.1 μM, FBR1B 0.5 μM, PR-F 0.05 μM, and RP-RB 0.25 μM. All the three systems contained a pair of primers that could amplify the gene of *Homo sapiens* ribonuclease P, which served as an internal standard. Amplifications were performed on a Veriti 96-Well Thermal Cycler PCR system (Applied Biosystems, USA) under the following conditions: 30 min at 50 °C; 2 min at 94 °C; 45 cycles of 20 s at 94 °C, 20 s at 52 °C, and 20 s at 72 °C; and a final extension of 5 min at 72 °C.

### Hybridization and signal detection

After amplification, the three reaction products derived from the same template were mixed into one tube (10 µl each). After 5 min of denaturation at 95 °C, the mixture was immediately placed on ice 5 min and then mixed with 5 μl of hybridization buffer (8× SSC, 0.6 % SDS, 10 % formylamine, and 10× Denhardt). A total of 10 μl of hybridization mixture was hybridized on the microarray for 1 h at 45 °C as described (Zhang et al. 2013). Then, the chip was washed for 30 s each with 1× SSC and 0.2 % SDS, 0.2× SSC, and 0.1× SSC at room temperature. Then the chip was incubated with 15 μl of streptavidin-horseradish peroxidase (Str-HRP, SIGMA) for 25 min at 37 °C. Subsequently, the chip was washed with PBST (phosphate buffer, 0.05 % Tween 20) 10 s at room temperature. Finally, 10 μL of pre-mixed CL HRP substrate luminol solution and H_2_O_2_ (Millipore Corporation, USA) was added to the chip and immediately detected with a micro-light level imaging system (developed in-house, China Patent Application No. 201310013267.X).

### Specificity and sensitivity

The specificity of this microarray was evaluated using 40 cultivated influenza A and B virus strains which were derived from CDC of Zhejiang Province and the National Institutes for Food and Drug Control. The genotype of these cultivated influenza viruses had been determined and cultivated at these two institutions. Influenza A and B Nucleic Acid Detection Kits (PCR-fluorescent probe) (Shenzhen Puruikang Biotech Co., Ltd.) which generally detected influenza A viruses and influenza B viruses, were used to compare results. A panel of non-influenza respiratory viruses, which include parainfluenza virus, adenovirus AD3, AD4, AD30, AD40, measles virus, mumps virus, and respiratory syncytial viruses HK6 and B, were also used to determine the specificity of the microarray.

In order to evaluate the sensitivity of this microarray in the detection of various subtypes of influenza virus, the RNA extraction of cultivated virus strains of 5 subtypes of influenza A virus and influenza B virus were serially diluted in tenfold increments and detected using the microarray. Influenza A and B Nucleic Acid Detection Kits (PCR-fluorescent probe) (Shenzhen Puruikang Biotech Co., Ltd.) were also used to compare results. They amplify the conserved M gene of influenza A viruses and the NS gene region of influenza B viruses.

### Clinical samples

To investigate the efficiency of clinical testing, 66 clinical swab samples were collected from the 307th Hospital of the Chinese People’s Liberation Army. Total RNAs were extracted using a TIANamp Virus RNA Kit as described in the manufacturer’s protocol. Then the clinical swab samples were detected using a microarray and verified using four different kinds of real-time RT-PCR kits. These real-time RT-PCR kits included a 2009 Influenza A Virus (H1N1) Nucleic Acid Detection Kit (PCR-fluorescent probe) (Shenzhen Puruikang Biotech Co., Ltd.), 2009 Influenza A Virus (H1N1) Nucleic Acid Detection Kit (PCR-fluorescent probe) (DAAN Gene Co., Ltd.), Seasonal Influenza A Virus (H3) Nucleic Acid Detection Kit (PCR-Fluorescent probe) (DAAN Gene Co., Ltd.), and Diagnosis Kit for H7N9 Avian Influenza Virus RNA (PCR-fluorescent probe) (Shenzhen Puruikang Biotech Co., Ltd.). These real-time RT-PCR kits identified and determined the concentration of HA gene of 2009 influenza A (H1N1), NA gene of 2009 Influenza A Virus (H1N1), influenza A (H3N2), and avian influenza H7N9 virus. They were all used as described in the manufacturer’s protocol.

## Results

### Primers and probes

#### Specificity of the microarray

**Fig. 2:**
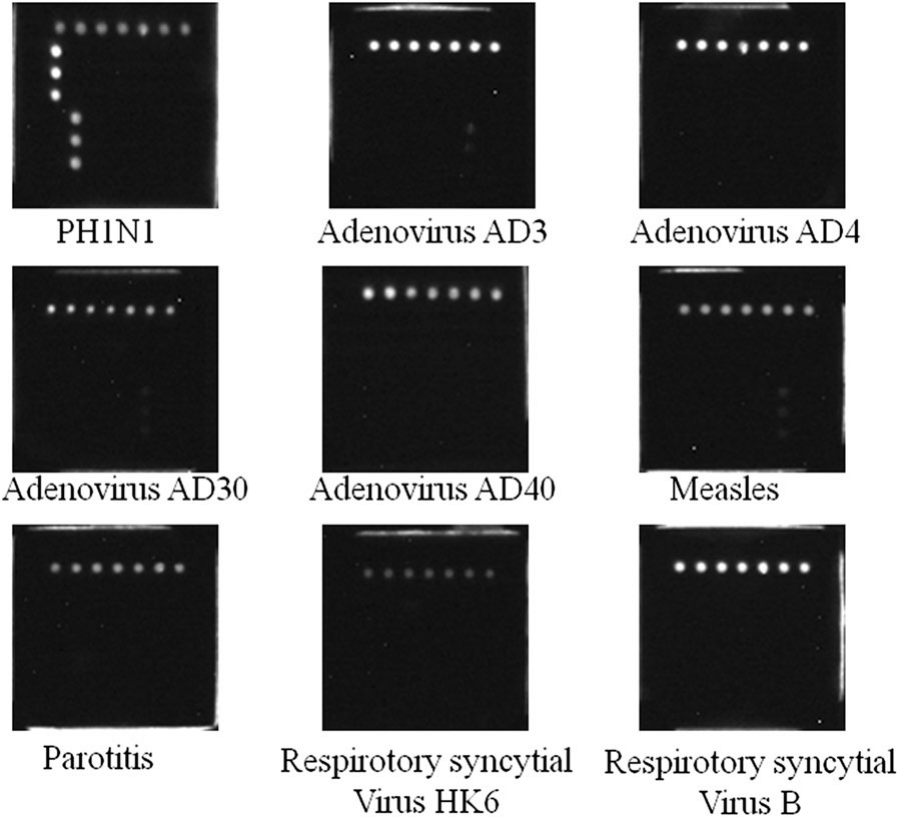
Detection of non-influenza respiratory viruses. The negative microarray results of these common respiratory viruses also demonstrated the specificity of this assay

In this assay, a total of 40 cultivated influenza virus strains were tested to determine the specificity of this microarray. The types or subtypes of these strains included 6 avian influenza A (H7N9), 6 influenza A (H3N2), 4 seasonal influenza A (H1N1), 2 2009 influenza A (H1N1), 18 avian influenza A (H5N1), and 4 influenza B virus. The results showed that one strain of H3N2 was mixed with H1N1, one strain of H5N1 was mixed with H1N1, another strain of H5N1 was mixed with H3N2, one strain of influenza B was mixed with H1N1, and one strain of H3N2 did not subtype because of its low concentration. The four mixed samples were confirmed by amplifying the M gene (for influenza A viruses) or NS gene (for influenza B viruses) of influenza using universal primers of influenza A and B viruses (Table 1) respectively and ligating the amplifications into the T-vector which were subsequently Sanger sequenced (data not shown). The Sanger sequencing results were coordinated to that of a microarray (results not shown). The results indicated that the microarray was able to distinguish these subtypes of influenza A and B viruses (Fig. 1c). There were no cross-signals between various influenza A virus subtypes, and the microarray simultaneously detected mixed subtypes in some mixed samples. Furthermore, because we observed a lower signal for H5N1 than the other subtypes with the general influenza A primers targeting the M gene (Fig. 1c), we investigated the number of mismatches of our probes to the H5N1 strains used here. Overall, there were 0, 1 and 1 mismatches in the forward primer, probe and reverse primer regions. In comparison, the suggested universal WHO primers (WHO 2009) also targeting the M gene have 1, 0 and 1 mismatches against the same strains in the forward primer, probe and reverse primer regions, respectively. In addition, the negative microarray results of these common respiratory viruses also demonstrated the specificity of this assay (Fig. 2). The information and detection results of the forty influenza virus strains are shown in Table 3.

**Table 3:** Statistics and detection of 40 influenza virus stains

Strain informations^a^	Microarray results^b^	Real time PCR results^c^	Cultivate results
Clinical isolated strain	H7 + N9 + A	20.3/22.2	H7N9
Clinical isolated strain	H7 + N9 + A	20.3/28.7	H7N9
Clinical isolated strain	H7 + N9 + A	18.4/26	H7N9
Clinical isolated strain	H7 + N9 + A	21.1/28.1	H7N9
Clinical isolated strain	H7 + N9 + A	26/28.3	H7N9
Clinical isolated strain	H7 + N9 + A	33/33	H7N9
BV/GuangdongLuohu/15/2007	B	20.33	B
B/Jiangxixinjian/39/2008	B + H1N1 + A	21.4	B
BY/FujianXinluo/54/2006	B	20.25	B
B/Tianjin/2/2001	B	18.06	B
A/Hiroshima/52/2005	H3N2 + A	19.3	H3N2
A3/Yunnan/1145/2005	H3N2 + A	19.3	H3N2
A3/Hanfang/359/1995	H3N2 + A	21.37	H3N2
A/Wisconsin/62/2005	H3N2 + A	19.91	H3N2
A/Minfang/1411/2002	A	–	H3N2
A3/Shenzhen/1/1999	H1N1 + H3N2 + A	21.22	H3N2
A/Newcaledonia/20/99	H1N1 + A	19.54	H1N1
A1/HubeiHongshan/53/2005	H1N1 + A	19.52	H1N1
A/Guangdongluohu/219/2006	H1N1 + A	20	H1N1
A1/Hufang/7/1999	H1N1 + A	18.96	H1N1
A/Beijin/SWL5/09	PH1N1 + A	20.42	H1N1/2009
A/Califonia/07/09	PH1N1 + A	20.84	H1N1/2009
A/Sichuan/1/2006 A1	H5N1 + A	30.89	H5N1
A/Sichuan/1/2006 A2	H5N1 + A	30.64	H5N1
A/Sichuan/2/2006 B	H5N1 + A	28.82	H5N1
A/Sichuan/3/06	H5N1 + A	28.32	H5N1
A/XJ/1/06 M1	H5N1 + A	26.93	H5N1
A/XJ/1/06 M2	H5N1 + A	26.55	H5N1
A/XJ/1/2006 L	H5N1 + A	27.04	H5N1
A/Anhui/1/2006 C	H5N1 + A	27.04	H5N1
Clinical isolated strain	H5N1 +H1N1 + A	28.3	H5N1
Clinical isolated strain	H5N1 + A	28.24	H5N1
Clinical isolated strain	H5N1 +H3N2 + A	25.59	H5N1
Clinical isolated strain	H5N1 + A	27.46	H5N1
Clinical isolated strain	H5N1 + A	32.19	H5N1
Clinical isolated strain	H5N1 + A	27.7	H5N1
Clinical isolated strain	H5N1 + A	32.08	H5N1
Clinical isolated strain	H5N1 + A	28.59	H5N1
A/GX/LA/13/04 K1	H5N1 + A	28.84	H5N1
A/GX/LA/13/04 K2	H5N1 + A	29.2	H5N1

^a^Six avian influenza A (H7N9) viruses were collected from Centers for Disease Control (CDC) of Zhejiang Province. The others were collected from the National Institutes for Food and Drug Control

^b^The results of microarray were corresponding combination signals of microarray probes. All results were obtained in 2/2 experiments

^c^The real-time PCR reagents for influenza A and B viruses were influenza A virus and B virus Nucleic Acid Detection Kits (PCR-fluorescent probe) (Shenzhen Puruikang Biotech Co., Ltd.)

#### The limit of detection of the microarray

**Fig. 3:**
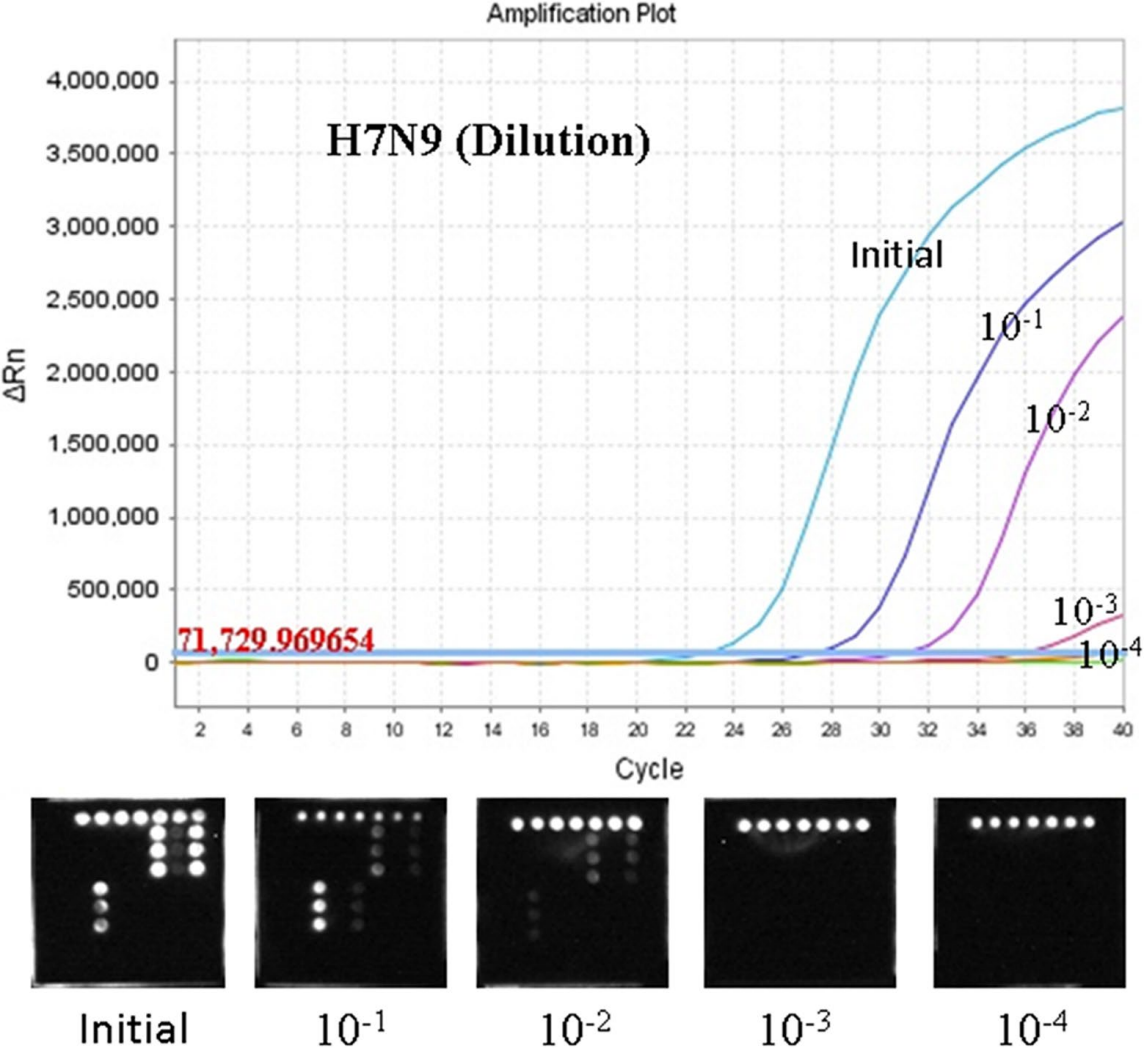
LOD of detection of avian influenza A (H7N9). The RNA extraction of avian influenza (H7N9) was serially diluted in tenfold increments and detected using this microarray and influenza A virus Nucleic Acid Detection Kits (PCR-fluorescent probe) (Shenzhen Puruikang Biotech Co., Ltd.). The microarray possessed a tenfold higher LOD to avian influenza A (H7N9) than the real-time RT-PCR method

To assess sensitivity, the microarray detection methods were compared to real-time RT-PCR. Results showed that the microarray have similar (for PH1N1) or tenfold higher limit of detection (LOD) (to the other subtypes of influenza A virus and influenza B virus) than the referenced real-time RT-PCR method. The LOD of general influenza A virus detection was lower than the LOD of genotype detection. The LOD for the two comparator real-time PCR assays was 1 × 10^3^ PFU/ml, so the LOD of the microarray was 1 × 10^3^–10 × 10^3^ PFU/ml. The comparative results of LOD of avian influenza A (H7N9) are shown in Fig. 3. The comparative results of LOD of other influenza viruses are shown in Table 4.

**Table 4:** The limit of detection of the microarray

Virus	Dilution	Micorarray results	Real time PCR results (Ct)	Virus	Dilution	Micorarray results	Real time PCR results (Ct)
H7N9	Original	H7 + N9 + A	23.7	H1N1	Original	H1N1 + A	14.97
	10^−1^	H7 + N9 + A	28.22		10^−1^	H1N1 + A	17.49
	10^−2^	H7 + N9 + A	31.79		10^−2^	H1N1 + A	21.55
	10^−3^	–	36.69		10^−3^	H1N1 + A	26.21
	10^−4^	–	–		10^−4^	H1N1 + A	30.78
	10^−5^	–	–		10^−5^	A	33.79
H5N1	Original	H5N1 + A	22.55	H3N2	Original	H3N2 + A	15.7
	10^−1^	H5N1 + A	27.98		10^−1^	H3N2 + A	18.68
	10^−2^	–	35.99		10^−2^	H3N2 + A	22.72
	10^−3^	–	–		10^−3^	H3N2 + A	27.08
	10^−4^	–	–		10^−4^	H3N2 + A	31.01
	10^−5^	–	–		10^−5^	–	33.29
PH1N1	Original	PH1N1 + A	16.24	B	Original	B	18.41
	10^−1^	PH1N1 + A	19.41		10^−1^	B	22.53
	10^−2^	PH1N1 + A	23.3		10^−2^	B	26.92
	10^−3^	PH1N1 + A	27.52		10^−3^	B	31.01
	10^−4^	PH1N1 + A	32.72		10^−4^	–	36.56
	10^−5^	–	–		10^−5^	–	–

The real-time PCR reagents for influenza A and B viruses were influenza A virus and B virus Nucleic Acid Detection Kits (PCR-fluorescent probe) (Shenzhen Puruikang Biotech Co., Ltd.). The microarray possessed similar or tenfold higher LOD than the real-time RT-PCR method. The reference PFU given for the PCR kits are approximated 1 × 10^3^ PFU/ml given by the manufacturers and vary between the kits

#### Detection of subtypes of cultivated virus and clinical positive swab samples

Sixty-six clinical swab samples were detected by microarray. Results indicated that 25 of the samples were 2009 influenza A (H1N1), 3 were influenza A (H3N2), 26 were influenza A, 1 contained both 2009 influenza A (H1N1) and influenza A (H3N2), and 11 were negative. Four different real-time PCR influenza genotyping kits served as reference methods. Results are shown in Table 5. Some samples that were only shown to contain only influenza A by microarray were confirmed to have specific subtypes using real-time PCR kits. This may be because the microarrays are less sensitive than real-time PCR kits. The results showed that microarray always failed to determine the subtypes of samples when the CT value of real-time RT-PCR exceeded 31. However, general detection of influenza A virus is possible even when the CT values were as high as 39. Consequently, general detection of influenza A virus was more sensitive than genotyping and detection of influenza A virus subtypes.

**Table 5:** The genotyping results of 66 clinical throat swab samples

No.	H1^a^ (Ct)	N1^b^ (Ct)	H3^c^ (Ct)	H7^d^ (Ct)	Microarray results	No.	H1 (Ct)	N1 (Ct)	H3 (Ct)	H7 (Ct)	Microarray results
1	27.79	29.59	–	–	PH1N1 + A	34	32.71	33.01	–	–	A
2	–	–	39.20	–	A	35	21.11	21.14	–	–	PH1N1 + A
3	–	–	34.26	–	A	36	21.08	22.59	–	–	PH1N1 + A
4	26.04	29.06	–	–	PH1N1 + A	37	29.17	31.4	–	–	PH1N1 + A
5	–	–	37.23	–	A	38	–	–	27.30	–	H3N2 + A
6	–	–	32.57	–	H3N2 + A	39	–	–	–	–	–
7	23.23	25.41	–	–	PH1N1 + A	40	30.06	31.83	–	–	PH1N1 + A
8	26.74	28.78	–	–	PH1N1 + A	41	25.34	21.22	–	–	PH1N1 + A
9	–	–	31.15	–	A	42	32.88	34.43	–	–	A
10	21.06	22.65	–	–	PH1N1 + A	43	20.37	21.41	–	–	PH1N1 + A
11	17.43	19.43	–	–	PH1N1 + A	44	26.09	26.3	–	–	PH1N1 + A
12	–	–	31.91	–	H3N2 + A	45	–	–	31.53	–	A
13	–	30.29	–	–	A	46	32.59	33.09	–	–	–
14	–	–	–	–	A	47	26.88	27.87	–	–	PH1N1 + A
15	25.79	26.51	–	–	PH1N1 + A	48	–	–	38.43	–	A
16	35.71	36.76	–	–	–	49	–	–	–	–	–
17	30.20	33.15	–	–	PH1N1 + A	50	31.75	31.37	–	–	PH1N1 + A
18	28.48	28.42	–	–	PH1N1 + A	51	–	–	35.09	–	A
19	27.42	26.41	33.61	–	PH1N1 + A	52	30.63	31.68	–	–	A
20	28.91	31.06	30.64	–	PH1N1 + H3N2 + A	53	35.10	35.15	–	–	–
21	27.97	29.61	–	–	PH1N1 + A	54	–	–	36.08	–	A
22	–	–	–	–	A	55	–	–	38.69	–	A
23	–	–	–	–	–	56	–	–	–	–	A
24	27.43	27.58	–	–	PH1N1 + A	57	–	–	–	–	–
25	–	–	–	–	A	58	–	–	–	–	–
26	26.63	28.38	–	–	PH1N1 + A	59	–	–	33.94	–	A
27	28.53	30	–	–	PH1N1 + A	60	–	–	–	–	–
28	35.71	35.16	–	–	–	61	27.38	27.57	–	–	PH1N1 + A
29	–	–	38.22	–	A	62	32.67	35.06	–	–	A
30	23.68	26.25	–	–	PH1N1 + A	63	24.28	21.9	–	–	PH1N1 + A
31	33.90	35.31	–	–	A	64	33.17	32.83	–	–	–
32	33.76	34.12	–	–	A	65	32.53	32.64	–	–	A
33	33.82	35.42	–	–	A	66	30.55	31.76	–	–	A

^a^The real-time PCR reference reagent of H1 was a 2009 Influenza A Virus (H1N1) Nucleic Acid Detection Kit (PCR-Fluorescent probe) (Shenzhen Puruikang Biotech Co., Ltd.)

^b^The real-time PCR reference reagent of N1 was 2009 Influenza A Virus (H1N1) Nucleic Acid Detection Kit (PCR-fluorescent probe) (DAAN Gene Co., Ltd.)

^c^The real time PCR reference reagent of H3 was Seasonal Influenza A Virus (H3) Nucleic Acid Detection Kit (PCR-Fluorescent probe) (DAAN Gene Co., Ltd.)

^d^ The real time PCR reference reagent of H7 was Diagnosis Kit for H7N9 Avian Influenza Virus RNA (PCR-fluorescent probe) (Shenzhen Puruikang Biotech Co., Ltd.)

Results indicated that 25 of the samples were 2009 influenza A (H1N1), 3 were influenza A (H3N2), 26 were influenza A, 1 contained both 2009 influenza A (H1N1) and influenza A (H3N2), and 11 were negative. The results showed that microarray always failed to determine the subtypes of samples when the CT value of real-time RT-PCR exceeded 31. However, general detection of influenza A virus is possible even when the CT values were as high as 39. Consequently, general detection of influenza A virus was more sensitive than genotyping and detection of influenza A virus subtypes

The statistical data of 66 clinical throat swab samples are shown in Table 6. The sensitivity, specificity, positive predictive values, and negative predicative values of microarray were 91.1, 60.0, 92.7, and 54.5 % those of the corresponding PCR kits. Furthermore, the value of genotyping for positive samples using this microarray was 53.6 %. The low specificity was attributable to the fact that there were four samples shown to contain influenza A by microarray and whose subtypes were not confirmed using real-time PCR kits. The results for these four specimens may be attributable to the low viral load or perhaps the influenza viruses were from subtypes not included in the microarray or real-time PCR kits. The low negative predicative values were attributable to the low sensitivity of the microarray relative to real-time PCR kits, which has been confirmed in cultivated influenza virus strains.

**Table 6:** The statistics of 66 clinical throat swab samples

Microarray	Real-time PCR	
	Positive	Negative
Positive (genotype)	51 (30)	4 (0)
Negative (genotype)	5 (0)	6 (0)

The sensitivity of microarray compared to real-time PCR was 91.1 %

The specificity of microarray compared to real-time PCR was 60.0 %

The positive predictive values of microarray compared to real-time PCR was 92.7 %

The negative predicative values of microarray compared to real-time PCR was 54.5 %

The diagnose accordance rate of microarray compared to real-time PCR was 86.4 %

The percent of genotyping for positive sample of microarray was 53.6 %

## Discussion

Influenza A virus can infect various host species, and the greatest diversity of subtypes is found in wildfowl. The influenza virus genome comprises 8 independent RNA strands, so it can easily undergo antigenic drift, antigenic shift, and reassortment, which allows it to cause major pandemics (Labella and Merel 2013). Over the past 100 years, four subtypes of influenza A virus have had an enormous impact on human beings, H1N1, H3N2, H2N2, and H5N1. Data from the American Center for Disease Control and Prevention have shown the influenza B virus to account for only 14 % of cases from October 2011 to May 2012 (CDC 2012). In 2013, there was a sudden human avian influenza A (H7N9) outbreak, inducing great panic in China. Clinical data suggested that H7N9 caused pneumonia with various degrees of severity, acute lung injury, acute respiratory distress syndrome, and even multiple organ failure. Increased concentrations of C reactive protein were found to be closely associated with mortality (Lu et al. 2014). According to published reports, the median age of patients who died of H7N9 was significantly higher than that of patients with nonfatal cases. Patients found to have increased risk of death upon admission were subjected to cheat imaging and found to have either bilateral lung inflammation or pulmonary consolidation. There were found to be significant levels of lymphopenia and decreases in oxygenation index in patients who eventually died (Ji et al. 2014).

Since October 2013, the strains of influenza A circulating in China have been completely resistant to amantadine drugs. Some of the 2009 influenza A (H1N1) strains have been found to be associated with lower susceptibility to NA inhibitor as indicated by weekly monitoring. Many studies have shown that the severity of the infection is more likely to be reduced if an NA inhibitor is administered within 48 h of the onset of influenza symptoms than when it is administered later (Hayden et al. 1997; McGeer et al. 2007; Monto et al. 1999). Vaccination is another effective way to prevent the spread of pandemic virus and reduce the severity of the disease (Luke and Subbarao 2006). Synthetic DNA vaccines and virus-like-particle vaccines have been developed for avian influenza A (H7N9) (Dormitzer et al. 2013; Fries et al. 2013; Klausberger et al. 2014; Smith et al. 2013; Yan et al. 2014). Because vaccine strains match circulating strains closely, it is important to rapidly and continuously monitor of epidemic influenza virus strains to determine which strains should be covered by seasonal influenza vaccinations (Bandt et al. 2012; Luke and Subbarao 2006; Subbarao et al. 2006). Rapid and accurate diagnosis of avian influenza A (H7N9) and other common influenza viruses is essential to improving clinical patient management and epidemiological investigation.

Currently, real-time RT-PCR is the most widely used molecular diagnostic approach to the detection of influenza virus in clinical settings (Choi et al. 2013; Dawood et al. 2009; Gao et al. 2013a; Hackett et al. 2014; Poon et al. 2009; Templeton et al. 2004). Although the real-time RT-PCR approach is generally more sensitive than microarray, only a few subtypes of influenza can be detected in the same reaction (Choi et al. 2013; Kuo et al. 2014). Microarrays are high-throughput and can be used to simultaneously detect several or even all the subtypes of influenza (Gall et al. 2009; Han et al. 2008; Heil et al. 2010; Ryabinin et al. 2011). However, traditional microarray detection requires expensive fluorescence scanners, which limit its use. Analytical CL is a versatile, sensitive method of detection with a wide range of uses, including enzyme-linked immunosorbent assays (Liu et al. 2012; Maiolini et al. 2013), lateral flow immunoassays (Maiolini et al. 2013; Wolter et al. 2008; Wutz et al. 2013), flow-through hybridization assays (Hommatsu et al. 2013), capillary electrophoresis (Jiang et al. 2013), flow injection CL analysis (Tan and Song 2013), and magnetic bead-based DNA hybridization assays (Li and He 2009). This is the first paper to subtype influenza viruses using a chemiluminescent oligonucleotide microarray. In this assay, a sensitive CL method that relies on horseradish peroxidase was used to catalyze the luminol-H_2_O_2_ for a conventional oligonucleotide microarray. A proprietary potable CL imaging system was established. The imaging system was based on CCD camera imaging technology and equipped with a power supply suitable for portable use (China Patent Application No. 201310013267.X). Furthermore, other commercial CL imagers based on CCD imaging technology (e.g. Amersham Imager 600, GE Healthcare Life Sciences) could also be used for this CL microarray. The new CL imager had a lower cost and a much faster detection speed than our previous visual microarray system which was based on quantum dot-catalyzed silver deposition. The cost of this CL imaging microarray was also lower than that of our previous fluorescence based microarray system (Zhang et al. 2013).

This assay was designed based on the following ideas: (1) priority was given to the detection avian influenza A (H7N9) (H7 and N9 were detected simultaneously). (2) Other than avian influenza A (H7N9), only the most common subtypes of influenza virus were detected by genotyping in this microarray. (3) Other than genotyping detection, the microarray also universal detected the more conservative M gene of influenza A viruses and NS gene of influenza B viruses to extend the range of the application. (4) One-step RT-PCR amplification was used to reduce the length of the experimental procedure and so reduce the risk of contamination. (5) Human-derived primers and probes served as internal standards; they were added to all the amplification reactions to monitor extraction of clinical samples, RT-PCR amplification, and microarray hybridization. (6) Low-cost CL imaging technology was used for detection in the microarray. This may allow this system to be used on a large scale.

A low-density CL oligonucleotide microarray was developed for genotyping detection of the newest avian influenza A (H7N9), avian influenza A (H5N1), 2009 influenza A (H1N1), seasonal influenza A (H1N1), and influenza A (H3N2). Influenza A viruses and influenza B viruses were also generally detected using this microarray. The microarray kits used here were capable of rapid, high-throughput, highly accurate readings, and the entire detection time from sample extraction to the production of genotyping results was 6–8 h. Six avian influenza A (H7N9) culture viruses and thirty-four other influenza culture viruses served as positive references to confirm the specificity and sensitivity of the microarray. Some non-influenza respiratory viruses were also tested using the microarray to further confirm this specificity. All the results showed that the microarray accurately detected the genotypes of these influenza viruses and of some mixed influenza strains. The LOD of genotyping and universal detection of influenza viruses were similar to or ten-fold higher than reference values for influenza real-time RT-PCR kits. Sixty-six clinical swab samples were assessed using microarray to determine the efficiency of this type of clinical testing and epidemiology. Results showed that the specificity and sensitivity of the microarray met the needs of clinical and epidemiological studies of the influenza virus.

This method has some limitations. RT-PCR amplification was here divided into three groups to amplify different subtypes of influenza viruses. This complicated the operation. However, reducing the number of amplification systems (increasing the number of primers in one amplification system) may have decreased sensitivity. Here, 11 pairs of primers were divided into three groups of amplification systems, and the LOD of this microarray was still ten times higher than that of the real-time PCR kits used for reference (the sensitivity of 2009 influenza A virus was similar to that of real-time RT-PCR kit). The 66 clinical swab samples were detected and microarray failed to show subtypes of samples when the CT value of real-time RT-PCR more than 32. However, general detection of influenza A virus was still positive even when the CT values were as high as 39. Consequently, the sensitivity of this CL detection strategy and microarray could be improved by further optimization. Moreover, avian influenza A (H7N9) and (H5N1) were not actually comparatively tested using the developed microarray and PCR assays due to the deficiency of clinical specimens.

Furthermore, the use of this strategy was restricted because of the limitations of detection for only novel avian influenza A (H7N9) and some of the most common human influenza viruses. The other influenza A subtypes such as H5N6 and H9N2, can not be typed. Thus, sequencing or other diagnostic methods are still needed to determine the exact subtypes of those influenza A viruses.

## Conclusions

A reliable CL detection oligonucleotide microarray was developed to genotype and detected avian influenza A (H7N9), avian influenza A (H5N1), 2009 influenza A (H1N1), seasonal influenza A (H1N1), and seasonal influenza A (H3N2). Influenza A viruses and influenza B viruses were also generally detected using this microarray. The results of detection of 40 cultivated influenza virus strains showed the microarray to be able to distinguish the subtypes of these influenza viruses very well. The microarray possessed similar or tenfold higher LOD than the real-time RT-PCR method. Sixty-six clinical swab samples were detected by microarray and verified using real-time RT-PCR to evaluate the efficiency of this microarray for clinical testing.

## Abbreviations

CDC: the Centers for Disease Control
CL: chemiluminescence
HA: hemagglutinin
LOD: limit of detection
M gene: matrix protein gene
NA: neuraminidase
NS gene: nonstructural protein gene
Str-HRP: streptavidin-horseradish peroxidase
